# *In vivo* myosin step-size from zebrafish skeletal muscle

**DOI:** 10.1098/rsob.160075

**Published:** 2016-05-25

**Authors:** Thomas P. Burghardt, Katalin Ajtai, Xiaojing Sun, Naoko Takubo, Yihua Wang

**Affiliations:** 1 Department of Biochemistry and Molecular Biology, Mayo Clinic Rochester, Rochester, MN 55905, USA; 2 Department of Physiology and Biomedical Engineering, Mayo Clinic Rochester, Rochester, MN 55905, USA

**Keywords:** single myosin detection *in vivo*, highly inclined thin illumination, transgenic zebrafish skeletal muscle, strychnine induced contraction, zebrafish skeletal myosin powerstroke, zebrafish skeletal myosin step-size

## Abstract

Muscle myosins transduce ATP free energy into actin displacement to power contraction. *In vivo*, myosin side chains are modified post-translationally under native conditions, potentially impacting function. Single myosin detection provides the ‘bottom-up’ myosin characterization probing basic mechanisms without ambiguities inherent to ensemble observation. Macroscopic muscle physiological experimentation provides the definitive ‘top-down’ phenotype characterizations that are the concerns in translational medicine. *In vivo* single myosin detection in muscle from zebrafish embryo models for human muscle fulfils ambitions for both bottom-up and top-down experimentation. A photoactivatable green fluorescent protein (GFP)-tagged myosin light chain expressed in transgenic zebrafish skeletal muscle specifically modifies the myosin lever-arm. Strychnine induces the simultaneous contraction of the bilateral tail muscles in a live embryo, causing them to be isometric while active. Highly inclined thin illumination excites the GFP tag of single lever-arms and its super-resolution orientation is measured from an active isometric muscle over a time sequence covering many transduction cycles. Consecutive frame lever-arm angular displacement converts to step-size by its product with the estimated lever-arm length. About 17% of the active myosin steps that fall between 2 and 7 nm are implicated as powerstrokes because they are beyond displacements detected from either relaxed or ATP-depleted (rigor) muscle.

## 1. Introduction

Myosin in muscle transduces ATP free energy into the work of moving actin. It is the muscle mover composed of a motor domain transducer containing ATP and actin binding sites, and, a lever-arm domain mechanically coupling motor impulsive force to the myosin filament backbone for transduction/mechanical-coupling. The lever-arm domain contains two stabilizing light chains, regulatory (RLC) and essential (ELC), and undergoes cyclical rotary movement to convert torque generated in the motor into actin linear displacement known as step-size. Actin displacement takes place against resisting load, placing shear strain on the lever-arm complex. The lever-arm bound RLC and ELC affect lever-arm stability [1–4] and impact motor strain sensitivity [5]. Motor and lever-arm domains of myosin assembled in the muscle sarcomere are the myosin cross-bridge.

Myosin translates actin when it is strongly bound. Qdot-labelled actin in the *in vitro* motility assay (Qdot assay) tracks unitary actomyosin encounters that are characterized with super-resolution microscopy. We verified that skeletal myosin has a dominant unitary step-size of approximately 5 nm [6,7] and discovered that cardiac myosin has three distinct unitary step-sizes (nominally 3, 5 and 8 nm) that move actin with varying relative step-frequencies adapting contractile power to changing demand [8]. Unitary step-size and step-frequency, modulated by the N-terminal binding of the cardiac ELC to actin [9], are myosin's principal structural characteristics relating to mechanical function. They are modified in nature to adapt the highly conserved myosin mover to its various tasks in muscle contractility.

The human ventricular RLC (MYL2), tagged with green fluorescent protein (GFP) at its C-terminus, was exchanged into permeabilized skeletal [10] or cardiac papillary muscle fibres [11]. In both cases, full recovery of isometric contraction from extensively and specifically exchanged myosins in the fibres implied the GFP did not deter contraction. The photo-activatable variant, RLC-PAGFP, was individually photoactivated in exchanged papillary muscle fibres isolating single myosins *in situ*. Single myosin lever-arm orientation was measured at super-resolution from fibres in rigor, relaxation and active isometric conditions to investigate lever-arm orientation and mechanical properties versus physiological state. Other experiments probed how heart disease-implicated mutants in the RLC affected these single myosin mechanical characteristics *in situ* [12]. We adapted this novel approach to *in vivo* experimentation in transgenic zebrafish embryos where we measured single myosin lever-arm orientation in relaxed skeletal muscle [13]. Single molecule emission patterns from relaxed muscle *in vivo* provided super-resolved dipole orientation constraints that were modelled using docking scenarios generated for the myosin head domain (subfragment 1 or S1) and GFP crystal structures [14,15] to estimate S1/GFP coordination. The S1/GFP coordination *in vivo* is rigid and the lever-arm orientation distribution well ordered in relaxed zebrafish muscle.

Normal zebrafish embryo swimming, displayed within 2 days of fertilization, is the alternating bilateral contraction of trunk skeletal muscle causing reciprocating tail movement propelling the embryo in water. An ‘accordion’ phenotype in a zebrafish embryo is attributed to the simultaneous (not alternating) contraction of the bilateral trunk muscles with treatment by strychnine [16]. Rather than swimming, these embryos have a quasi-static shortened trunk owing to the contraction that does not propel. Strychnine blocks the inhibitory glycine receptor (GlyR) to induce contraction in zebrafish and humans. Blocked GlyR causes skeletal muscle stiffness, because the muscle cannot relax for an extended period following spasm. In work described here, we use strychnine to induce isometric skeletal muscle contraction in zebrafish embryos. No effect owing to strychnine was observed on embryonic heart rate or blood circulation, and strychnine-induced isometric contraction was reversible.

Skeletal muscle *in vivo* in isometric contraction, relaxed and ATP-depleted (rigor) conditions were imaged at approximately 1 Hz frame rate to acquire a signal-to-noise ratio (S/N) sufficient to estimate single GFP dipole orientation at super-resolution. Dipole orientation converts to single lever-arm orientation using the known S1/GFP coordination [13]. Lever-arm orientation measured for sequential frames converts to step-size using the lever-arm length of approximately 8.5 nm estimated from the skeletal S1 crystal structure [15]. The modest acquisition rate might produce aliased real-time data of lever-arm rotation in isometric contraction; nonetheless, large sets of step-size amplitudes from the various muscle physiological states indicate that approximately 17% of the active myosin steps falling between 2 and 7 nm are powerstrokes because they are beyond displacements detected from relaxed muscle and muscle in rigor. Physiological controls measured isometric contraction from permeabilized transgenic zebrafish embryos, indicating the myosin GFP tag does not deter skeletal muscle contractility.

*In vitro* Qdot assay estimates of adult zebrafish skeletal myosin step-size resembles values measured for the *in vitro* cardiac myosin. Actin in the Qdot assay is unloaded, in contrast with the *in vivo* active isometric embryos, where step-size estimates are from the loaded myosin. *In vivo* and *in vitro* myosin step-size measurements compare a fundamental structural characteristic of the myosin mover. It defines *in vitro* and *in vivo* myosin complementarity, and relates definitively how context affects myosin function. *In vivo* single myosin detection is a new multi-scaled technology allowing one-to-one registration of selected myosin molecular characteristics with whole muscle phenotype.

## 2. Methods

### 2.1. *In vitro* biochemical assays and statistics

#### 2.1.1. Actin-activated myosin ATPase

Actin-activated myosin ATPase was measured as previously described [17]. Myosin at a final concentration of 0.5 µM was titrated with 1, 2, 5, 17, 30, 43, 99 and 102 µM actin. Inorganic phosphate measurements were performed using the Fiske & Subbarow [18] method.

Actin-activated ATPase results were parametrized using Michaelis–Menten kinetics,2.1
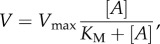
for *V* the ATPase rate equal to *V*_max_ at saturating actin concentration, actin binding constant *K*_M_ and actin concentration [*A*]. *V* versus [*A*] curves are fitted using a nonlinear algorithm to determine constants *V*_max_ and *K*_M_.

Experiments were conducted at room temperature (20–22°C).

#### 2.1.2. *In vitro* motility and Qdot assays

*In vitro* motility and Qdot assays were performed using total internal reflection fluorescence microscopy exactly as described [9]. Actin sliding velocities for the *in vitro* motility assay, *s*_m_, were quantitated using FIESTA software [19].

Frictional loading assays were performed as described in [20] with subtle modifications. Rhodamine labelling of actin filaments was performed with rhodamine–phalloidin and actin in a 1.2 : 1 molar ratio. The labelled actin moved over myosin adsorbed to the surface of a flow cell as described [7]. The flow cell was infused at the start with the mixture of myosin and α-actinin in concentrations of 0.16 µM myosin and 0–5 µg ml^−1^ α-actinin (Cytoskeleton, Denver, CO).

In Qdot assays, images were acquired with an EMCCD camera (Andor, Belfast, UK) at 50 ms intervals indicated by Δ*t* and using Andor's SOLIS software. Intensity values were converted to photons using the conversion formula in SOLIS and the images output in TIFF format for reading into ImageJ. We track movement of the Qdot-labelled actin at super-resolution using the ImageJ plugin QuickPALM [21].

All *in vitro* motility and Qdot assay experiments were conducted at room temperature (20–22°C).

#### 2.1.3. Qdot assay event–velocity histogram simulation

Change in Qdot position over two sequential frames is divided by Δ*t* to obtain velocity. The acquired Qdot-labelled actin velocities were binned to create the event-velocity histogram. In general, muscle myosins produce multiple unitary steps with differing step-sizes, *d_j_*, and relative step-frequency, *ω_j_*, for unitary step *j* [7,8].

*V*_max_ and average motility velocity, *s*_m_, are measured under saturating actin and myosin conditions, respectively. They are constant parameter inputs to the simulation that are characteristic to each myosin tested. Simulation approximates the Qdot motility event–velocity histogram in the low velocity domain of approximately 0–3 natural velocity units (vu) where (*d*_I_/Δ*t*) = 1 for *d*_I_ the intermediate step-size (near 5 nm) and where unitary events dominate. The unknown parameter set actively searched in the simulation consists of the actin binding probability for myosin (one free parameter), step-size (three free parameters) and relative step-frequency (3 – 1 = 2 free parameters owing to normalization). Trial parameter values are generated in the simulation by random choice from a broad range of values set at the start of the simulation by visual inspection of the data. The simulation is programmed in Mathematica (Wolfram Research, Champaign, IL).

Native zebrafish skeletal RLC (ZRLC) in purified adult skeletal myosin (Zmys) was replaced with GFP-tagged expressed human cardiac RLC (HCRLC-GFP), giving Zmys-GFP then used in the Qdot assay. A Qdot assay dataset consists of 10 and 13 acquisitions (one acquisition is one *in vitro* motility movie and corresponding event–velocity histogram) from preparations of one control Zmys and one unknown Zmys-GFP. Three separate Zmys and Zmys-GFP protein preparations were used giving a total of 30–39 acquisitions. Comparison of simulated curves to data uses the *χ*^2^ goodness-of-fit test that is weighted by event total then summed over all the acquisitions for evaluating global goodness of fit.

Simulated data ensembles were created by using the 30–39 best-fitting event–velocity histogram simulations generated for a Qdot assay dataset. The simulations are combined linearly to approximate the measured event–velocity histogram from the pooled data (when appropriate, see below) with coefficients greater than or equal to 0 while minimizing the *χ*^2^ goodness-of-fit test with all points equally weighted. This simulation method is identical to that described previously [9].

#### 2.1.4. Statistics

Statistical analysis tested whether data are distributed normally at the significance level of 0.05 and using several methods, including Cramer–von Mises, Pearson chi-squared and others.

*In vitro* motility experiments using Zmys and the Zmys-GFP preparations corresponded to 30–39 independent event–velocity histograms for each isoform. We simulated each experiment independently to estimate single myosin mechanical characteristics consisting of three step-sizes and three step-frequencies. We compared three step-sizes or three step-frequencies as categorical variables in factor one and the three independent datasets acquired for the separate Zmys or Zmys-GFP preparations (with 10–13 independent event-velocity histograms for each isoform) in factor 2 using two-way ANOVA with Bonferroni or Tukey post-tests for significance at the 0.05 level. This test indicated no significant difference in the independent Zmys or Zmys-GFP datasets, hence datasets were pooled.

*In vitro* step-size and step-frequency parameters from the Qdot assay were compared between Zmys and Zmys-GFP using one-way ANOVA with Bonferroni or Tukey's post-tests for significance at the 0.05 level. These outcomes are described in Results.

Tension data statistical methods are described in Results.

### 2.2. Zebrafish sample preparations

#### 2.2.1. Preparation of wild-type and exchanged zebrafish skeletal myosin

Two to six adult wild-type (WT or Zmys) zebrafish (11 months old, dominant leopa) were obtained from the Mayo Clinic Zebrafish Core Facility. Fish were anaesthetized for 3–5 min using tricaine methanesulfonate (MS-222, 168 mg l^−1^) then euthanized. The fish head, skin and organs were removed with forceps on ice. The trunk muscle (0.25–1.3 g) was dissected and homogenized at 4°C in tissue weight to 2× buffer volume (40 mM imidazole pH 7.2, 2 mM MgCl_2_, 1 mM DTT, 0.05 mg ml^−1^ leupeptin and 1 mM PMSF) and washed twice by pelleting in the Eppendorf microcentrifuge. Myosin extraction was done with 3× volume extraction buffer (0.3 M NaCl, 150 mM sodium phosphate pH 6.8, 10 mM sodium pyrophosphate, 10 mM MgCl_2_, 1 mM EGTA, 1 mM DTT, 0.05 mg ml^−1^ leupeptin, 0.01 mg ml^−1^ chymostatin and 0.05 mg ml^−1^ pepstatin). After 30 min stirring on ice, the homogenate was pelleted by spinning in the centrifuge. The supernatant containing the extracted myosin was further purified by low ionic strength precipitation by adding 12× volume ice cold 5 mM sodium phosphate buffer pH 6.5 with 1 mM DTT. Myosin was dissolved in 0.6 M NaCl, 50 mM Tris–HCl pH 7.5, 1 mM DTT, 0.05 mg ml^−1^ leupeptin, 1 mM EGTA) and further purified using ultracentrifugation (10^5^ *g* for 2 h at 4°C). The supernatant containing partially purified myosin was dissolved in 50% glycerol and stored in the freezer at −20°C. The extraction yielded 1.9–15 mg of myosin from two to six fish.

cDNA of WT human cardiac RLC (HCRLC) was a gift from Dr D. Szczesna-Cordary (University of Miami). HCRLC-GFP was constructed as previously described [10]. These expressed light chains were used to prepare the exchanged skeletal myosins.

Native ZRLC was replaced with expressed human cardiac RLC (HCRLC) or HCRLC-GFP (Zmys-HCRLC or Zmys-GFP) as described [3] with minor modifications. Briefly, native ZRLC was depleted from purified fish skeletal myosin extracted from adult fish by incubation of myosin with 1% Triton X-100 and 5 mM CDTA, pH 8.5 for 15 min at 23°C. The Triton/CDTA-treated myosin was then precipitated with 12 volumes of ice-cold water with 1 mM DTT for 1 h and was collected by centrifugation (16 000*g* for 30 min at 4°C). The missing RLC fraction was calculated from the SDS/PAGE of the treated myosin. The depleted myosin was dissolved in a buffer of 0.4 M KCl, 50 mM MOPS (pH 7.0), 2 mM MgCl_2_ and 1 mM DTT. The Zmys RLC reconstitution was done by adding HCRLC or HCRLC-GFP to depleted Zmys in a 3 : 1 molar ratio. The mixture was stirred slowly on ice for 2 h and then precipitated by overnight dialysis against 12 volumes of ice-cold 5 mM DTT and 10 µg ml^−1^ leupeptin. The precipitated Zmys-HCRLC or Zmys-GFP complexes were collected by centrifugation and resuspended in a buffer contained 0.6 M KCl, 50 mM MOPS (pH 7.0), 2 mM MgCl_2_, 2 mM DTT and 10 µg ml^−1^ leupeptin. The myosin solution was mixed with glycerol in a 1 : 1 (vol : vol) ratio and stored at −20°C.

#### 2.2.2. Protein extraction from zebrafish embryos

All protein extraction from zebrafish embryos was used to identify and quantitate myosin and myosin light chains using sodium dodecyl sulfate–polyacrylamide gel electrophoresis (SDS–PAGE). 10–20 WT or transgenic 6 days post-fertilization (dpf) embryos were anaesthetized with tricaine methanesulfonate (168 mg l^−1^) for 1–2 min, then euthanized. Embryo head and yolk were separated with forceps and the remaining tissue transferred to Eppendorf centrifuge tubes containing 40–80 µl^−1^ of Biorad gel sample buffer with 2% SDS. The samples were homogenized then cleared by centrifugation. Extracts were heated to 95°C for 3 min followed by a 30 min incubation at room temperature, then cleared by centrifugation. The procedure was repeated, and the supernatant of the last centrifugation step saved at −20°C for SDS–PAGE.

#### 2.2.3. Preparation of zebrafish embryo skeletal myosin

Preparation of myosin from euthanized embryos was identical to that from the adult fish described above except that the tissue came from 120 to 140 WT or transgenic embryos at 6 dpf.

#### 2.2.4. Transient expression of HCRLC-PAGFP in zebrafish embryos

All *in vivo* single myosin detection experiments used transiently expressed HCRLC-PAGFP as described previously [13]. Transiently expressed HCRLC-PAGFP specifically and efficiently labelled the zebrafish embryo skeletal myosin in a mosaic pattern in trunk muscle where the overall tagged myosin fraction in the muscle is below quantitation level using SDS–PAGE. Low RLC-PAGFP expression is suitable for the single myosin detection experiments to minimize background fluorescence levels.

#### 2.2.5. Transgenic animals expressing HCRLC-(PA)GFP

We coinjected transposase mRNA and plasmid containing a Tol2 construct with the zebrafish UNC-45b enhancer and the gene for HCRLC-(PA)GFP into the cytoplasm of one-cell-stage embryos. At 3–4 dpf, we screened embryos for green fluorescence excited by 405 nm transmitted light using a 10× objective. Embryos with striated GFP signal more uniformly covering the muscle were grown to maturity in the zebrafish core facility. After three months, the founder fish (F0) were screened by outcrossing with WT. The F1 embryo offspring were used for experiments and grown to maturity for an F2 generation.

Transgenic zebrafish had visible GFP expression throughout the trunk muscle arranged in the striated pattern of skeletal muscle similar to that within the fluorescent locations of the transiently expressed HCRLC-(PA)GFP [13]. Uniform expression in the muscle allows assessment of HCRLC-GFP-tagged myosin contractility and expression level. WT fish were incrossed as control, and F1 transgenic fish were outcrossed with WT producing F2 embryos. F2 transgenic GFP(+) or GFP(−) (TgGFP(+) or TgGFP(−)) embryos having strong or weak GFP expression in the trunk muscle were selected at 3–4 dpf.

Experiments were conducted at room temperature (20–22°C).

#### 2.2.6. Contractility in zebrafish embryos

WT, TgGFP(+) and TgGFP(−) embryos at 5–6 dpf were permeabilized exactly as described [22]. Permeabilized embryos were attached at the extreme ends of their trunk muscle to a transducer for measuring tension.

Tension measurements used relaxing solution containing (in mM) 29.3 K-propionate, 6.6 MgCl_2_, 0.17 CaCl_2_, 7 EGTA, 5 ATP, 20 phosphocreatine, 20 imidazole, 15 µg ml^−1^ creatine kinase, 1 DTT and 1 mg ml^−1^ leupeptin at pH 7.0. Activating solution contained 14.5 K-propionate, 6.4 MgCl_2_, 7.4 CaCl_2_, 7 EGTA, 5 ATP, 20 phosphocreatine, 20 imidazole, 15 µg ml^−1^ creatine kinase and 1 DTT at pH 7.0.

#### 2.2.7. Expression of HCRLC-GFP-tagged myosin in zebrafish embryos

The gene expression level of the native skeletal RLC (ZRLC) was estimated in F2 embryos using reverse transcription polymerase chain reaction (RT-PCR) experiments. RT-PCR mRNA expression profiles were determined for several relevant genes in WT, TgGFP(−) and TgGFP(+) embryos at 6 dpf to estimate the expression levels of ZRLC and GFP-tagged expressed human cardiac RLC (HCRLC-GFP). Total RNA was extracted from the F2 embryos using Trizol reagent (Invitrogen, Carlsbad, CA), the extracted RNA was purified using RNeasy mini kit (Qiagen), and total mRNA was quantified using a NanoDrop v. 2.0 spectrophotometer (Thermo Scientific, Waltham, MA). Complimentary DNA was synthesized from 1 µg of total RNA by using a Superscript III first-strand synthesis system (Invitrogen) primed with random hexamers according to the manufacturer's instruction. RT-PCR was performed using Platinum Taq DNA polymerase (Invitrogen) with gene specific primers to quantitate mRNA expression. Expression levels of ZRLC and HCRLC-GFP that are expected to be affected by transgenesis were normalized relative to the presumably unaffected skeletal myosin heavy chain (MHC; mhyz2) or β-actin genes.

ZRLC and HCRLC-GFP protein expression levels were measured using SDS–PAGE of expressed and extracted proteins. Quantities of purified embryonic myosin were too low to detect quantitatively on a Sypro Ruby-stained gel, hence we combined Sypro Ruby staining and protein immunoblotting. Purified WT adult zebrafish myosin and an *in vitro* expressed HCRLC standard were run on adjacent lanes on an SDS–PAGE gel that was stained with Sypro Ruby. Intensity for the ZRLC and HCRLC bands was compared to estimate ZRLC quantity. Protein bands from an identical gel were transferred to a membrane for Western blotting using a primary RLC antibody (10906-1-AP, Proteintech, Chicago, IL). Antibody staining of the known amounts of ZRLC and HCRLC calibrated the standardized immunoblotting protocol for their detection.

Myosin extracted from WT, TgGFP(−) and TgGFP(+) embryos were run on adjacent lanes on an SDS–PAGE gel, then the proteins were transferred to a membrane for Western blotting using the RLC antibody. Intensities of the HCRLC-GFP and ZRLC bands were converted to HCRLC-GFP and ZRLC amounts and combined into the replaced fraction, ZRLC_rep_, given by2.2
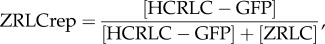
where […] indicates mole protein and all bands come from the TgGFP(+) embryos.

Independently, the amount of ZRLC removed was measured relative to β-actin (the loading standard) in WT and transgenic embryos and using the RLC and a β-actin antibody (Cell Signaling Technology, Danvers, MA). The ZRLC fraction lost owing to transgenesis, ZRLC_rem_, is given by2.3
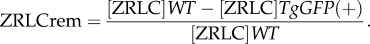


Experiments were conducted at room temperature (20–22°C).

#### 2.2.8. Strychnine treatment of tagged zebrafish embryos

We induced isometric contraction in 3–4 dpf embryos transiently expressing HCRLC-PAGFP using strychnine. Strychnine causes simultaneous activation of opposing bilateral skeletal trunk (tail) muscles inducing their isometric contraction as described by Hirata *et al*. [16]. A 10 mM stock strychnine solution was made fresh daily. Two 3 or 4 dpf embryos tagged with RLC-PAGFP were placed in separate 200 µm deep microfluidic channels with the channel side up and on one chip. Embryos were immersed in 20 µl of 30% Danieau buffer (D-buffer, 17.4 mM NaCl, 0.21 mM KCl, 0.12 mM MgSO_4_, 0.18 mM Ca(NO_3_)_2_, 1.5 mM HEPES, pH 7.6). Embryos were mechanically stimulated by touching with forceps (touch test) to elicit a normal escape reflex consisting of a short burst of tail strokes for swimming. We introduced 10 or 70 µM strychnine to the buffer of one embryo and performed touch-tests of control untreated and strychnine-treated embryos every 3–5 min thereafter. Untreated embryos showed a normal escape reflex to the touch-test over the course of the experiments. Strychnine-treated embryos did not respond to the touch test after 10 or 3 min but instead displayed a transient ‘shivering’ movement indicating apparent extended bilateral contraction in the trunk muscles.

After the 10 or 3 min incubation time, bathing solution volumes were drawn down to less than 6 µl, and the microfluidic was inverted, then placed on top of a #0 glass coverslip forming a water tight seal with the glass for imaging as described [13]. Treated and untreated embryos were alternatively imaged over time at a 0.5–1 Hz frame rate to collect single molecule emission images from photo-activated RLC-PAGFP. Imaging sessions were completed within approximately 10 min. Heart rate was measured visually in the microscope every 2–3 min and remained constant at approximately 120 beats per minute over the course of the experiment. Blood circulation in capillaries in the fish tail was also monitored every 2–3 min in the microscope by visualizing blood cell movement. Blood cell flow did not qualitatively change over the course of the experiment.

Following the imaging session, embryos were rinsed three times in 10 ml of D-buffer, then returned to separate 60 mm Petri dishes containing 10 ml of D-buffer without strychnine. Embryos treated with 10 µM strychnine recovered the normal touch test response compared with control with 10 min incubation in the D-buffer without strychnine. Embryos treated with 70 µM strychnine recovered the normal touch test response compared with control after less than 12 h incubation in the D-buffer without strychnine.

Recovered embryos with relaxed trunk muscles underwent a second imaging session to collect the single molecule emission images from the photo-activated RLC-PAGFP. Imaging sessions were again completed within 10 min.

Experiments were conducted at room temperature (20–22°C).

#### 2.2.9. Tagged zebrafish embryos in strong actin binding state

We prepared 3–4 dpf control untreated and strychnine-treated embryos just as described above. We used 70 µM strychnine for the treated embryo and occasionally tested the escape reflex of both control untreated and strychnine-treated embryos. Untreated embryos showed a normal escape reflex to the touch test over the course of the experiments. Strychnine-treated embryos did not respond to the touch test for the 10–120 min session after introduction of strychnine. No effect was observed on heart rate or blood circulation over the course of the experiment. We assume that MgATP is exhausted in the muscle after 90–120 min in isometric contraction induced by 70 µM strychnine. We refer to the ATP exhausted state as rigor. Sarcomere lengths shortened by approximately 10% (from approx. 2.1 to 1.9 µm) in the ATP exhausted muscle compared with control. We did not attempt to reverse the ATP-depleting strychnine treatment.

After the 90–120 min incubation time, treated and untreated embryos were alternately imaged over time at a 0.5–1 Hz frame rate to collect single molecule emission images from photo-activated RLC-PAGFP. Imaging sessions were completed within approximately 10 min.

### 2.3. *In vivo* single myosin orientation quantitation

#### 2.3.1. S1/GFP coordination

**Figure 1.**
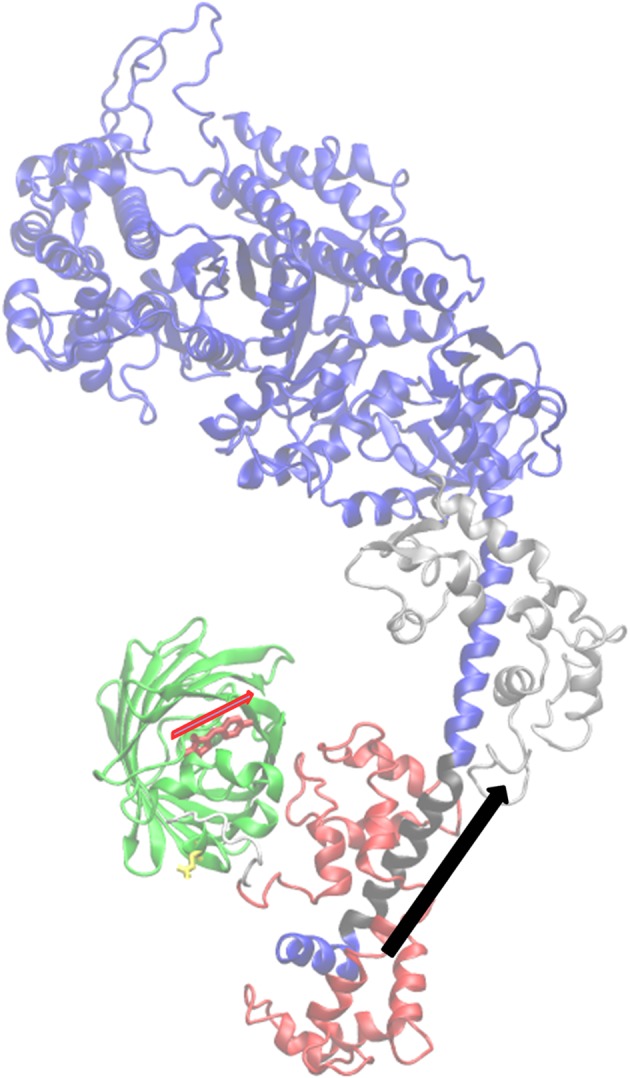
The coordination of the GFP moiety (green) and its emission dipole moment (red arrow) with myosin S1 consisting of a heavy chain (blue and black), ELC (silver) and RLC (red). The portion of the lever-arm in the heavy chain appearing in black is the α-helix segment associated with the lever-arm orientation and depicted by the black arrow.

Figure 1 indicates the MHC in blue and black. The RLC (red) and ELC (silver) bind the lever-arm of MHC. The GFP moiety is in green. The thick black arrow indicates the lever-arm α-helix symmetry axis where RLC binds (black section of the α-helix). The black arrow is related to the laboratory frame by coordinates (β,α), also referred to as lever-arm coordinates. The GFP chromophore in the middle of the β-barrel is indicated in red. The red arrow at the chromophore is the emission dipole orientation related to the laboratory frame by the coordinates (β′,α′) also referred to as probe coordinates. GFP is linked to the RLC C-terminus by the white linker. The S1 structure in figure 1 is that of human β-cardiac myosin from the homology modelling [23] of its sequence using the chicken skeletal myosin S1 crystal structure 2mys [15].

#### 2.3.2. Orientation super-resolution measured from tagged zebrafish muscle

Zebrafish embryos were confined to the microfluidic chamber described above constructed from polydimethylsiloxane. Single molecule fluorescence measurements from the photo-activated HCRLC-PAGFP-tagged myosin lever-arms were made on an inverted microscope using highly inclined thin illumination (HILO), as described previously [13].

In all fluorescence experiments, pump and observation exciting laser light polarization is p-polarized perpendicular to the fibre symmetry axis. A sparse population of probes is photo-activated to achieve the most selective orientation distribution of photo-activated probes by using the lowest practical pump beam intensity. We identified single molecule events by their quantized intensity change owing to photoactivation or photobleaching over time just as described [13]. Orientation super-resolution of 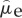 [*A*], the *emission* dipole moment of the activated single molecule, is determined by pattern recognition as described previously [13].

#### 2.3.3. S1/GFP coordination

The S1/GFP coordination of the zebrafish skeletal muscle was determined as described previously [13] and shown in figure 1. For this study and using the same method, we checked the new dipole orientation data for relaxed, rigor and active muscle (summarized subsequently in Results) for consistency with the S1/GFP coordination in figure 1. We found that the new data representing the wider set of muscle physiological states are consistent with the previous data in selecting the S1/GFP coordination in figure 1 over other docked models.

#### 2.3.4. *In vivo* step-size

**Figure 2.**
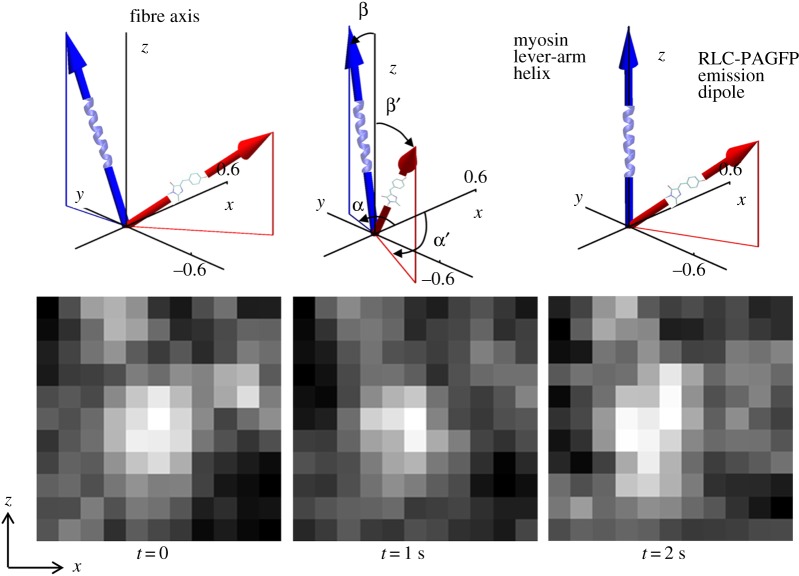
Time-resolved coordinates for GFP chromophore emission dipole moment in red and lever-arm helix in blue corresponding to spherical polar angles (β′,α′) and (β,α) defined relative to a fibre fixed frame (top row). The three reference frames depict the trajectories for a single dipole or lever-arm helix corresponding to the single myosin images in the bottom row. Images are intensity normalized and have 3.5–4.2 signal-to-noise (S/N) ratios.

Single molecule images provided super-resolved orientation of the photo-activated RLC-PAGFP emission dipole in zebrafish skeletal muscle. Dipole orientations were computed from images that were continuously recorded for approximately 2 min. Object spatial drift, if present, was removed by large frame image alignment prior to analysis. The time-resolved coordinates have dipole or lever-arm helix spherical polar angles (β′,α′) or (β,α) defined relative to a fibre fixed frame shown in figure 2 (top row). They are the trajectories for a single dipole or lever-arm helix corresponding to the single myosin images in figure 2 (bottom row).

**Figure 3.**
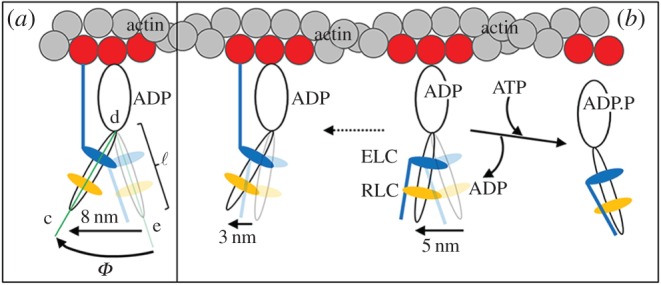
The N-terminal extension of skeletal ELC(A1) or cardiac ELC modifies myosin step-size in muscle. Muscle myosin can perform an 8 nm step shown in panel *a* or two other shorter steps of 5 or 3 nm depicted in panel *b*. The 5 nm step forgoes the ELC/actin interaction and is predominant in porcine cardiac and rabbit skeletal muscle. The 3 nm step is the rare separation of a full 8 nm step into two shorter steps, possibly owing to slow dissociation of ADP. We show here that adult zebrafish skeletal myosin uniquely has equal propensity for 5 and 8 nm steps. Panel *a* also indicates lever-arm rotation angle *Φ* from the angle formed from ∠cde and lever-arm length ℓ used in equation (2.4).

The arc subtended by *Φ*, the angle a single lever-arm helix rotates in sequential (time-correlated) images, defines a sequence of chords on a circle of diameter ℓ equal to the lever-arm length indicated in figure 3*a*. Chord length is step-size, *d*, given by2.4
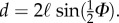

Step-sizes computed by equation (2.4) from many single myosin trajectories are summarized as a histogram of incremental step-lengths versus the number of observed events. Step-lengths distribute differently for various muscle physiological states. The strychnine-induced active state is steady-state contraction, but a single myosin cross-bridge is non-stationary in its time sequence as it cycles through lever-arm orientations for actin associated and disassociated conformations. Relaxed state from resting muscle has single myosins maintaining a mostly actin-dissociated form and dynamically averaged lever-arm orientation that is stationary in its time sequence over sampling intervals of 1–2 s. Strong actin-associated rigor state, induced in the muscle by MgATP depletion, is static (hence stationary in its time sequence) as single myosins maintain a characteristic lever-arm orientation at the low free energy end of the powerstroke.

Active myosin develops force with its lever-arm swing starting in the high free-energy actin-bound state of the myosin before ADP release (pre-powerstroke). Active myosin is also actin-dissociated (relaxed) and strongly actin-bound in rigor after ADP release. We model the step-size distribution in the active isometric state, *v*_ac_, as the linear combination of distributions composed from relaxed, *v*_re_, rigor, *v*_ri_, and the unknown force developing pre-powerstroke residual, Δ*v*, such that2.5
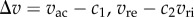
for unknown constant coefficients *c_j_*. The relaxed, rigor and active step-size histograms *v*_re_, *v*_ri_ and *v*_ac_ are the basis vectors covering probability space spanned by tagged lever-arm orientations. They have Poisson-distributed noise randomly sampled, whereas Δ*v* is minimized for each trial by selection of *c_j_* subject to constraints *c_i_* ≥ 0, *j* = 1, 2 using constrained linear programing in Mathematica. We estimate the mean and variance for Δ*v* at each point in the histogram from the family of residuals produced in the trials.

## 3. Results

### 3.1. Light chain exchanged Zmys

**Figure 4.**
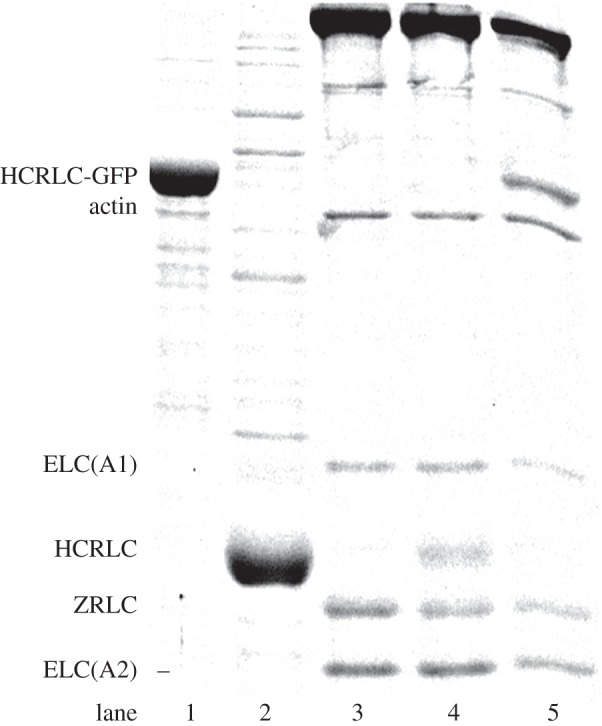
SDS–PAGE of expressed HCRLC-GFP (lane 1), expressed HCRLC (lane 2), Zmys (lane 3), HCRLC exchanged Zmys (lane 4) and HCRLC-GFP exchanged Zmys (lane 5). Quantitation indicates 70/30% ZRLC/HCRLC or ZRLC/HCRLC-GFP in the exchanged Zmys.

Figure 4 shows the SYPRO Ruby-stained SDS–PAGE gel for Zmys (lane 3), Zmys-GFP (lane 5) and Zmys-HCRLC (lane 4). All fluorescence intensities are normalized relative to the ELC(A1) value in each lane. Intensity quantitation shows exchange reduces ZRLC and increases HCRLC-GFP (lane 1) or HCRLC (lane 2) equally at approximately 30%, implying the exchanged myosins in the Zmys-GFP or Zmys-HCRLC preparations are heterogeneous with 70% ZRLC and 30% HCRLC-GFP or HCRLC. Adult zebrafish myosin has an ELC(A2)/ELC(A1) ratio of approximately 2. Electronic supplementary material, figure S1 compares the HCRLC-GFP exchange efficiencies for rabbit skeletal, Zmys, and porcine ventricular cardiac myosins.

### 3.2. Actin-activated ATPase of zebrafish skeletal myosin

**Figure 5.**
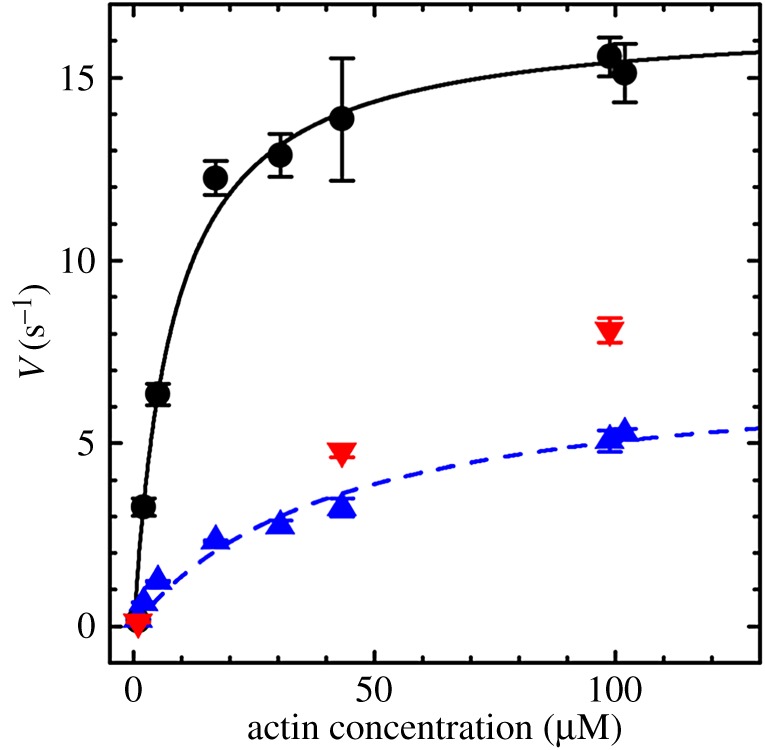
Actin-activated ATPase for Zmys (black circles), Zmys-GFP (blue up pointing triangles) and Zmys-HCRLC (red down pointing triangles) with error bars indicating standard deviation for four trials from two independent protein preparations (Zmys and Zmys-GFP) and two trials from one protein preparation (Zmys-HCRLC).

Figure 5 shows the actin-activated ATPase for Zmys (circles), Zmys-GFP (blue up triangles) and Zmys-HCRLC (red down triangles) all with error bars indicating standard deviation. Table 1 summarizes Michaelis–Menten parameters for the Zmys and Zmys-GFP. The actin-activated ATPase data for the Zmys-HCRLC sample are intermediate but similar to Zmys-GFP. The data show qualitatively that the GFP is not the primary cause of lowered enzymatic activity. Rather the HCRLC, the exchange process, or both cause lowered enzymatic activity.

**Table 1.** Zebrafish skeletal myosin actin-activated ATPase, *in vitro* motility and *in vitro* force.^a^

	Zmys	Zmys-GFP	Zmys-HCRLC
*V*_max_ (s^−1^)	16.7 ± 0.6	7.2 ± 0.8	—
*K*_M_ (μM)	8.2 ± 1.4	42.1 ± 11.3	—
*s*_m_ (µm s^−1^)^b^	1.39 ± 0.17	1.52 ± 0.16	—
isometric force^c^ (μg ml^−1^)	3.81 ± 0.42	2.50 ± 0.17	2.63 ± 0.22

^a^*V*_max_ and *K*_M_ are defined in equation (2.1). All experimental errors indicated are standard deviation for 3 replicates (30–39 for *s*_m_).

^b^Motility velocity, *s*_m_, is from the Qdot motility assay with myosin bulk concentration of 0.16 µM.

^c^In units of α-actinin concentration from data in figure 6.

### 3.3. *In vitro* motility

*In vitro* motility velocity, *s*_m_, for Zmys and Zmys-GFP versus myosin bulk concentration show velocities saturate at approximately 0.1 µM bulk concentration and converge to similar maximum velocities. The percentage of moving actin filaments is 70–80% for both samples, and for both rhodamine and Qdot-labelled actin. Motility velocities for myosin bulk concentrations of 0.16 µM are summarized in table 1.

### 3.4. *In vitro* force measurements

**Figure 6.**
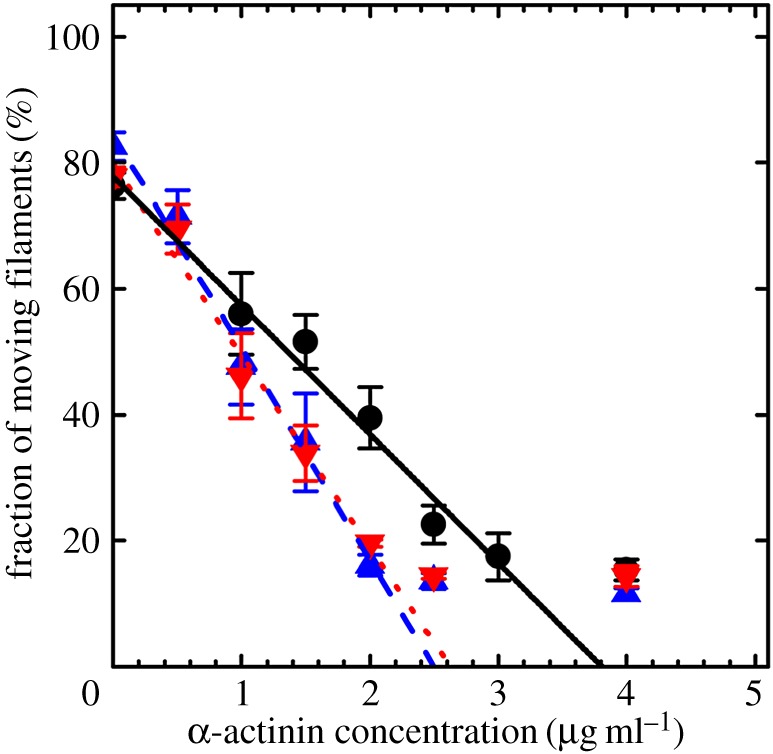
The percentage fraction of moving actin filaments versus α-actinin concentration for Zmys (black circles), Zmys-GFP (blue up triangles) and Zmys-HCRLC (red down triangles). Error bars indicate standard deviation for data from more than 1000 actin filaments at each α-actinin concentration and from three independent protein preparations. Lines extrapolated to the *x*-intercept indicate relative isometric force. Data at the highest α-actinin concentrations where linearity fails are excluded from the fits, because α-actinin is unable to fully inhibit actin filament sliding.

Figure 6 shows the fraction of moving actin filaments versus α-actinin concentration in the motility assay for Zmys (circles), Zmys-GFP (blue up triangles) and Zmys-HCRLC (red down triangles) with error bars indicating standard deviation. Data extrapolates to isometric contraction (*x*-intercept) conditions using a fitted line excluding data at the highest α-actinin concentrations where linearity fails, because α-actinin is unable to fully inhibit actin filament sliding [20]. Fitted lines are computed using the generalized linear model for the Gaussian-distributed data and 95% confidence level. Linear fits for Zmys (solid line), Zmys-GFP (blue long-dashed line) and Zmys-HCRLC (red short-dashed line) are shown in figure 6 and the force parameters (*x*-intercept) summarized in table 1. Isometric force is modestly reduced by a factor of approximately 0.7 compared with Zmys owing to exchange of either HCRLC (Zmys-HCRLC) or HCRLC-GFP (Zmys-GFP), whereas there is no significant difference between Zmys-HCRLC and Zmys-GFP. Alternatively, filament velocity can be the dependent variable in the assay [24]. The latter gives equivalent results for the isometric force.

### 3.5. Qdot assay

**Figure 7.**
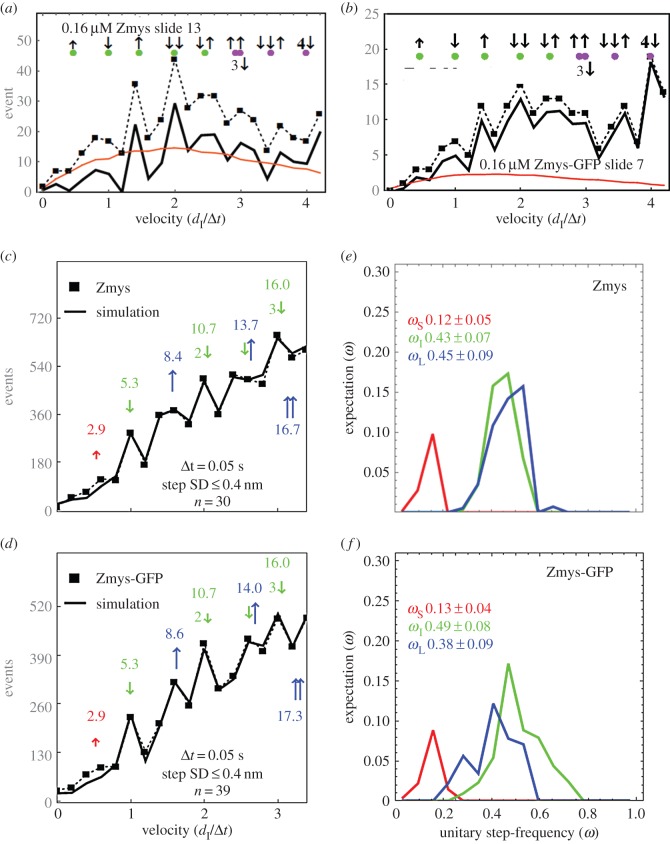
Raw Qdot assay event-velocity histogram data (panels *a*,*b*) indicating a dataset from one acquisition of actin gliding data (solid square and dashed line), baseline of thermal/mechanical fluctuations (red line) and their difference (solid black line). Green markers near the top of the panels indicate the positions of the dominant step types with the first three peaks corresponding to short (short up arrow), intermediate (long down arrow) and long (long up arrow) unitary steps. Green and blue dots indicate the position of the assigned unitary steps or unitary step combinations as indicated. Qdot assay event-velocity histograms (panels *c*,*d*) and the unitary step-frequency expectations (panels *e*,*f*) for pooled data from adult zebrafish skeletal myosins are also shown. Myosin isoforms are the native (Zmys) and exchanged (Zmys-GFP) proteins. The event-velocity histogram shows the low-velocity end with unitary step-size data (solid square and dashed line made up of pooled data from 30 or 39 acquisitions for the Zmys and Zmys-GFP isoforms) and simulation (solid line). Step-sizes correspond to the short (↑red), intermediate (↓ green) and long (↑ blue) steps, with associated numeric values in nm. Step-frequency expectations correspond to the short (red), intermediate (green) and long (blue) step-sizes, with numeric mean values *ω*_S_, *ω*_I_ and *ω*_L_ ± s.d.

The Qdot assay has labelled actin filaments approximately 1 µm long translating over surface-bound zebrafish skeletal myosin. Figure 7*a,b* shows raw Qdot assay event-velocity histogram data from one acquisition with Qdot-labelled actin gliding over Zmys and Zmys-GFP. The event-velocity histogram covers the low velocity domain of approximately 0–4 natural velocity units (vu) where (*d*_I_/Δ*t*) = 1 for *d*_I_ = 5.77 and 5.05 nm (Zmys and Zmys-GFP) and frame capture interval Δ*t* = 50 ms. Raw data (solid square connected with dashed line) and the baseline contribution from thermal/mechanical fluctuations (red solid line) are subtracted to give net motility owing to myosin activity. Large green markers near the top of the panels indicate the positions of the dominant step types with the first three peaks corresponding to short (short up arrow), intermediate (long down arrow) and long (long up arrow) unitary steps. The short up arrow appears only once, because its low probability prevents its detectable contribution in combination with other step-sizes. Green and blue markers indicate the position of the assigned unitary steps or unitary step combinations. The principal basis for accurate simulation is identifying positions and probabilities for the five dominant unitary or unitary combination step-types indicated by green markers.

Panels *c* and *d* show baseline subtracted event-velocity histograms of pooled data from 30 and 39 acquisitions from control Zmys and Zmys-GFP. Panels *e* and *f* show the step-frequency expectation for Zmys and Zmys-GFP. The Zmys-GFP sample is heterogeneous with HCRLC-GFP replacing 30% of the native ZRLC. Pooled data (solid square connected with dashed line) and simulation (solid line) are shown for the event–velocity curves. Peaks or inflection points appearing below 2 vu are short (↑red or S), intermediate (↓green or I) and long (↑_L_ blue or L) step-sizes in nm. Steps in combination and step-size standard deviations are shown. One-way ANOVA test indicates the *d*_L_ for Zmys-GFP is significantly longer than the native length. The modest level of exchange in the Zmys-GFP suggests the step-size difference between the native and modified cross-bridge is larger than 0.2 nm and owing to the presence of the GFP tag.

Panels *e* and *f* show the step-frequency (*ω*) expectations, expectation values and standard deviations for the S, I and L unitary steps. The expectation curves indicate the relative probability for step-frequency values along the abscissa. The area under the colour-coded curves for the S, I and L steps equals expectation values *ω*_S_, *ω*_I_ and *ω*_L_, respectively. The sum *ω*_S__+_ *ω*_I__+_ *ω*_L_ = 1 for each myosin species. The Zmys step-frequencies differ from any previously studied myosin species, with the *ω*_L_ larger, *ω*_I_ smaller and *ω*_S_ unchanged from cardiac myosin [7]. The HCRLC-GFP exchange returns step-frequencies to their values in native cardiac myosin. The effect of HCRLC-GFP exchange on I and L step-frequencies is significant using the one-way ANOVA test. The modest level of exchange in the Zmys-GFP suggests the step-frequency discrepancies between the native and modified cross-bridges are larger and owing to the presence of the GFP tag.

Data in figure 7 indicate that the zebrafish skeletal myosin moves actin with three unitary step-sizes similar to that observed for cardiac myosin. Other recent work attributes much of the long step prevalence in cardiac myosin to the interaction of the cardiac ELC (vELC) N-terminus extension with actin by the model indicated in figure 3 [9]. Unlike cardiac myosin that contains only the vELC, adult Zmys has mixed isoforms for ELC with and without the N-terminus extension (A1 and A2 isoforms) in an A2/A1 ratio of approximately 2 (figure 4).

Zmys *in vitro* characteristics (motility velocity, isometric force and step-size) are largely unaffected, whereas bulk characteristics (actin-activated ATPase) are affected, by the HCRLC or HCRLC-GFP exchange. *In vitro* motility experiments have a second dead-head removal step following myosin adsorption to the nitrocellulose surface that is impossible for the actin-activated ATPase assay. It apparently removes more of the zebrafish myosin denatured by the light chain exchange conditions.

### 3.6. Effect of HCRLC-GFP on permeabilized zebrafish skeletal muscle contractility

**Figure 8.**
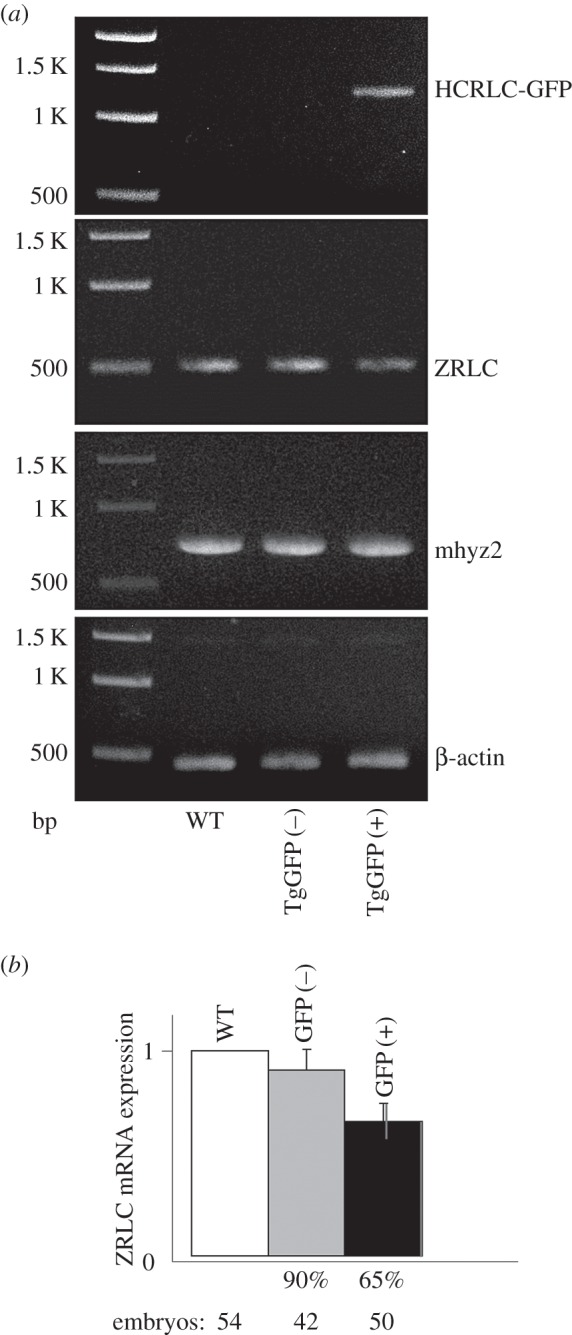
mRNA levels in a 1% agarose gel (panel *a*) for ZRLC, HCRLC-GFP, skeletal myosin heavy chain (mhyz2) and β-actin measured using RT-PCR on WT, TgGFP(−) and TgGFP(+) embryos and the quantitation (panel *b*) comparing ZRLC mRNA levels normalized by β-actin intensity and reported as relative to the WT level. Levels for mhyz2 and β-actin mRNA expression are controls. Quantitation in panel *b* used four replicates on different embryo sets. Error bars show standard deviation for total embryos indicated below the bar charts.

Figure 8*a* shows a 1% agarose gel indicating mRNA levels measured for ZRLC, HCRLC-GFP, skeletal myosin heavy chain (mhyz2) and β-actin using RT-PCR on WT, TgGFP(−) and TgGFP(+) embryos. Expression levels of the mhyz2 and β-actin are used for gel loading controls. RT-PCR quantitation (like that in panel *a*) of data from four independent embryo preparations indicate HCRLC-GFP and ZRLC expression are anti-correlated (Pearson correlation −0.91), and that ZRLC expression declines in TgGFP(−) and TgGFP(+) embryos by 10 and 35 ± 9% (panel *b*). In panel *b*, error bars show standard deviation for total embryos indicated below the bar charts. Anti-correlation of HCRLC-GFP and ZRLC expression suggests approximately 35% of the ZRLC is removed (equation (2.3)) from TgGFP(+) embryos.

**Figure 9.**
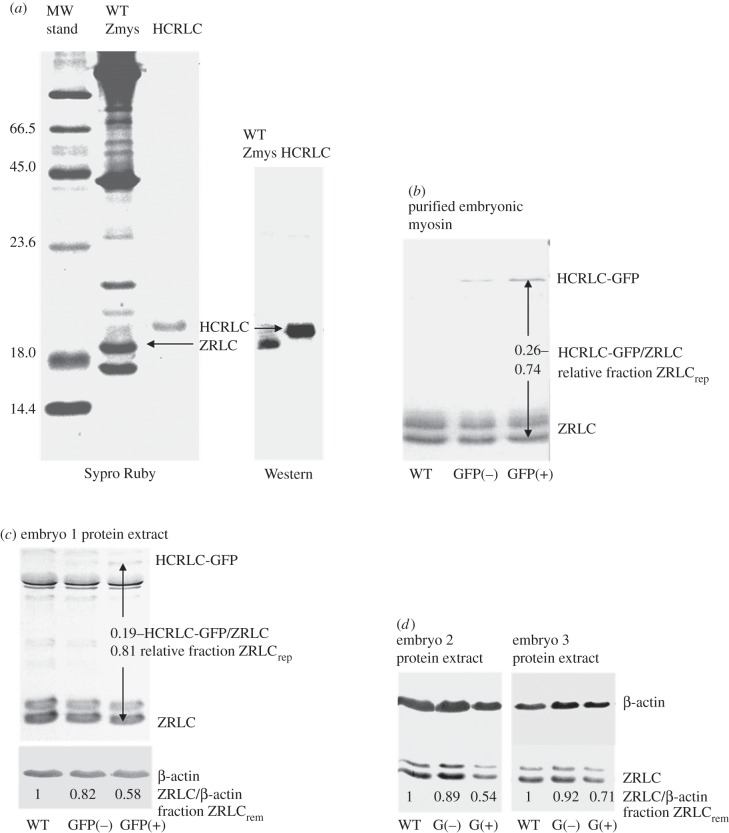
Sypro Ruby and immunoblots (Western) SDS–PAGE gels compare protein standard HCRLC and proteins extracted from adult or embryonic zebrafish. Immunoblot primary antibodies are for RLC (HCRLC and ZRLC) or β-actin. TgGFP(+/−) embryo samples are denoted by GFP(+/−) or G(+/−). Panel *a* shows partially purified adult WT zebrafish myosin (WT Zmys) and *in vitro* expressed HCRLC. The latter has concentration measured with the Bradford assay (Biorad, Hercules CA). Sypro Ruby staining of both samples determines the ZRLC content in the WT Zmys sample. The Western of the same samples calibrates immunoblot intensities for HCRLC and ZRLC when using the primary RLC antibody. Panel *b* shows the immunoblots for purified skeletal myosin from WT, TgGFP(−) and TgGFP(+) zebrafish embryos detecting the unknown expression levels of HCRLC-GFP and ZRLC. Relative mole fraction of HCRLC-GFP (ZRLC_rep_) is computed using equation (2.2) in the text. A second experiment with the purified myosin provided a ZRLC_rep_ = 0.25 (data not shown). Panel *c* indicates Western blots from an embryo protein extract detecting HCRLC and β-actin. ZRLC intensities are normalized relative to β-actin expression and compared across the WT, TgGFP(−) and TgGFP(+) zebrafish. The relative intensities measure the loss of ZRLC coincident with gain in HCRLC-GFP expression due to the transgenesis. The ZRLC fraction lost (ZRLC_rem_) is quantitated using equation (2.3) in the text. Detection of the expressed HCRLC-GFP in the protein extract is also indicated, permitting calculation of ZRLC_rep_. HCRLC-GFP and β-actin positions in the gel overlap. Panel *d* indicates Western blots from embryo protein extracts. Like panel *c*, these samples indicate the relative loss in ZRLC with the gain in HCRLC-GFP expression in the transgenic zebrafish.

Alternatively, we detected the ZRLC and HCRLC-GFP protein content in WT, TgGFP(−) and TgGFP(+) embryos using SDS–PAGE and combining Sypro Ruby staining and immunoblotting for detection of protein content. Figure 9 shows the Sypro Ruby-stained and immunoblotted SDS–PAGE gels. The Sypro Ruby gel band intensities establish ZRLC content in a purified adult zebrafish myosin relative to a known amount of *in vitro* expressed HCRLC (panel *a*). These samples produce calibrated blot intensities for HCRLC and ZRLC under a standardized protein immunoblotting protocol using the RLC antibody described in Methods. The standards are compared with immunoblots from HCRLC-GFP and ZRLC in WT, TgGFP(−) and TgGFP(+) embryos (panel *b*) to measure relative fractions of HCRLC-GFP and ZRLC content (ZRLC_rep_, equation (2.2)). Additional experiments in panels *c* and *d* estimate ZRLC_rep_ (panel *c*) and establish the relative amount of ZRLC removed from WT, TgGFP(−) and TgGFP(+) embryos (ZRLC_rem_, equation (2.3), panels *c* and *d*) using embryo protein extracts. Western blots of embryo protein extracts quantitated ZRLC_rem_ relative to a β-actin loading control as described in Methods. Results are summarized in table 2.

**Table 2.** Fractional HCRLC-GFP replacement of ZRLC (ZRLC_rep_) and ZRLC removal (ZRLC_rem_) in zebrafish embryos.^a^

	ZRLC_rep_	ZRLC_rem_
TgGFP(+)	0.23 ± 0.04	0.38 ± 0.08

^a^Fractions computed using equations (2.2) and (2.3) showing mean and standard deviation for 3 or more replicates. The table summarizes ZRLC_rep_ data from figure 9*b–d* (just two of three replicate measurements of ZRLC_rep_ are shown in the figure) and ZRLC_rem_ data from figure 8 (four replicates from RT-PCR data) and figure 9 panels *c, d* (the three replicates shown).

Table 3 summarizes the results of the tension measurements on permeabilized embryos. Tension indicates no significant difference between WT, TgGFP(−) and TgGFP(+) embryos using the one-way ANOVA test, suggesting GFP-tagged and native myosin cross-bridges produce similar isometric force. We tested tension data significance assuming isometric force produced by a GFP-tagged cross-bridge in TgGFP(+) is related linearly to the native cross-bridge force, so that the ensemble force from a TgGFP(+) embryo is given by3.1
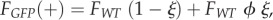
where *F*_WT_ and *F*_GFP(+)_ are ensemble isometric forces measured for WT and TgGFP(+) embryos, *ϕ* is the ratio of isometric forces for a GFP-tagged and native cross-bridge, and *ξ* is the tagged cross-bridge fraction in TgGFP(+). Data in table 2 suggest 0.19 ≤ *ξ* ≤ 0.46 where *ξ*s are distributed normally, and error is standard deviation. Measured isometric forces summarized in table 3 are likewise normally distributed and have the standard deviations given there. We computed *F*_GFP(+)_ estimates (c*F*_GFP(+)_) using equation (3.1) for mean *ξ*, 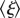, varying from 0.19 to 0.46, and by replacing *ϕ* with its random variates.

**Table 3.** Permeabilized zebrafish trunk muscle contractility in force per unit area.^a^

	tension	*n*
WT	17.0 ± 8.0	32
TgGFP(−)	14.2 ± 7.8	30
TgGFP(+)	14.5 ± 8.5	29

^a^Mean tensions and standard deviations measured in kN m^−2^ (kilonewtons per metre squared) for *n* embryos.

The *ϕ* variates are generated from equation (3.1) solved for *ϕ*,3.2
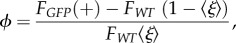
by substituting all experimental values for *F*_WT_ and *F*_GFP(+)_ while eliminating sample *ϕ*s < 0. Sample *ϕ*-data histogram, mean and standard deviation give the prior distribution and inverse Gaussian likelihood function (using the mean and standard deviation) for the Bayes posterior *ϕ*-distribution. Random variates for the posterior *ϕ*-distribution are on the domain 0 < *ϕ* < ∞ as appropriate for *ϕ*-data. We qualified cF_GFP(+)_ estimates for *F*_GFP(+)_ using mean, variance and normal distribution testing, then estimated *χ*^2^ goodness of fit using the squared difference between histograms of c*F*_GFP(+)_ and *F*_GFP(+)_ over the bin size of 2 kN m^−2^ to locate best choices for 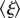 and 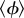 in the TgGFP(+) embryos.

**Figure 10.**
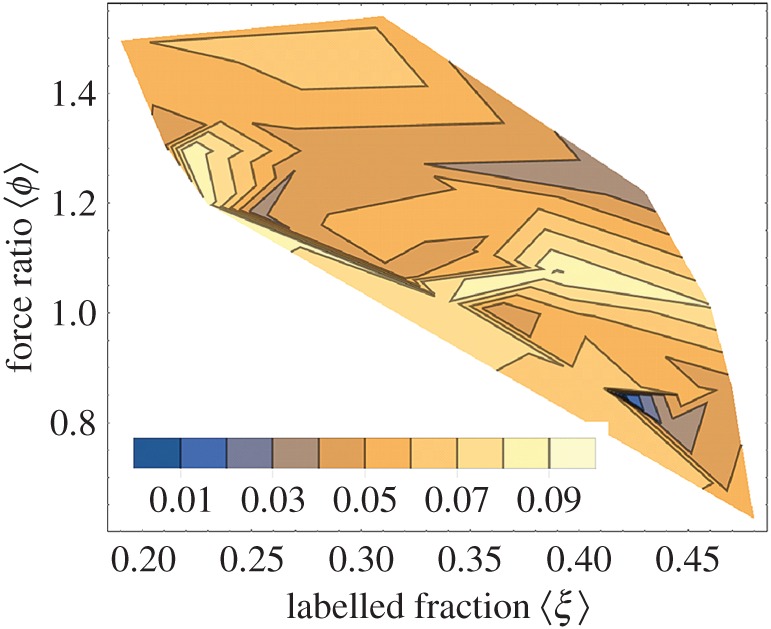
Contour plot of *χ*^2^ for the c*F*_GFP(+)_ approximation of *F*_GFP(+)_ indicating the best-fitting average labelled fraction 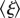 and force ratio 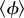 relating the isometric forces of tagged and native cross-bridges (see equation (3.1)). The *χ*^2^ minimum is indicated in deep blue and according to the legend.

Figure 10 shows the contour plot for *χ*^2^ versus (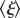, 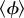). Agreement of c*F*_GFP(+)_ estimates for F_GFP(+)_ are indicated by the deep blue region for 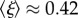 and 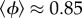. These findings combine and interpret the RT-PCR and SDS–PAGE estimates for ZRLC removal and replacement by HCRLC-GFP in TgGFP(+) embryos and the isometric tension data (table 3) under the assumption implied by equation (3.1). They also conform to the *in vitro* force data assessment that the GFP tag causes little change in isometric force.

### 3.7. *In vivo* step-size of zebrafish skeletal myosin

**Figure 11.**
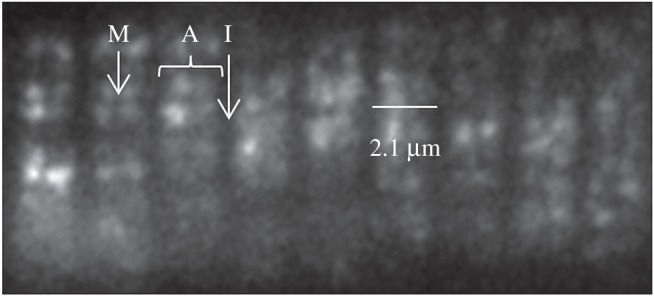
Strychnine-treated zebrafish embryo trunk muscle *in vivo* and under HILO illumination. The 3–4 dpf zebrafish embryo is transiently expressing HCRLC-PAGFP and showing the characteristic striated pattern of myosin in the thick filament of skeletal muscle. Bright points of light are emission from photo-activated PAGFP, some of which are single myosins. M-line, A-band and I-band are identified by the many unphotoactivated chromophores in the sarcomeres that emit with low efficiency owing to their small extinction coefficient for the 488 nm excitation light. Bar indicates sarcomere length.

Figure 11 shows a frame from a movie of active zebrafish embryo trunk muscle *in vivo* and under HILO illumination. The 3–4 dpf zebrafish embryo is transiently expressing HCRLC-PAGFP. Electronic supplementary material, figure S2 indicates several examples of single molecule events from active muscle identified by their quantized activation over background intensity followed by quantized photobleaching back to background. Electronic supplementary material, figure S2 also identifies the files containing extended image sequences of single PAGFP-tagged myosin *in vivo* from active trunk muscle. The sequence in figure 2 (bottom) is taken from one of these extended sequences. Electronic supplementary material, figure S3 is identical to electronic supplementary material, figure S2 except for relaxed muscle.

**Figure 12.**
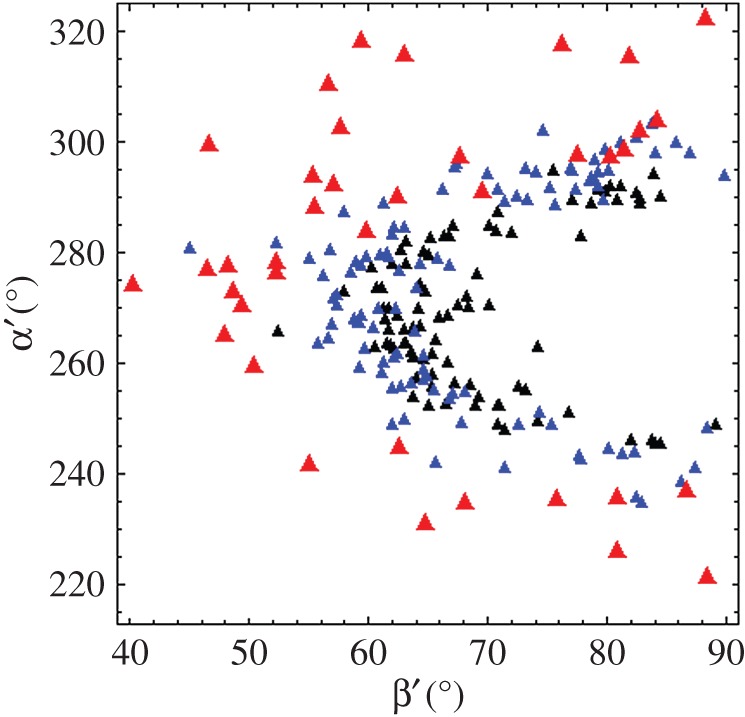
PAGFP dipole spherical polar angles (β′,α′) defined in figure 2 from single myosins in active isometric (red), relaxed (blue) and rigor (black) muscle.

Single molecule intensity patterns from zebrafish embryo skeletal muscle myosins were fitted using the pattern recognition algorithm and subjected to orientation super-resolution analysis [25,26]. Time-resolved image sequences were acquired from relaxed, active isometric and rigor muscle just as described in Methods. Figure 12 contrasts characteristic PAGFP dipole spherical polar angles (β′,α′) from single myosins in active (red), relaxed (blue) and rigor (black) muscle. Single myosins in rigor have dipole moments confined to the smallest radius arc around the (β′,α′) = (90 270) point. Active single myosins maintain dipole moments appearing at the largest radius arc around the (β′,α′) = (90 270) point. Relaxed myosin dipoles fill in the area at intermediate radii. Active myosins also appear in relaxed and rigor regions. Data in figure 12 represent dipole orientation data for relaxed, rigor and active muscle impacting the best choice for the S1/GFP coordination. The new data representing the wider set of muscle physiological states are consistent with the previous data selecting (over other docked models) the S1/GFP coordination in figure 1 [13].

Preferred dipole orientations are apparent in the figure 12 scatter plot. These data reflect lattice constraints and molecular crowding effects that impact all physiological states. Other more complex scenarios are also likely to explain dipole orientation distribution. For instance, two conformations for cross-bridges in a myosin dimer are indicated by blocked and free S1s in EM reconstructions of smooth muscle myosin heavy meromyosin [27] and cardiac myosin thick filaments [28,29]. This appears to be a general myosin regulation mechanism [30] implicit in the dipole distributions depicted in figure 12.

**Figure 13.**
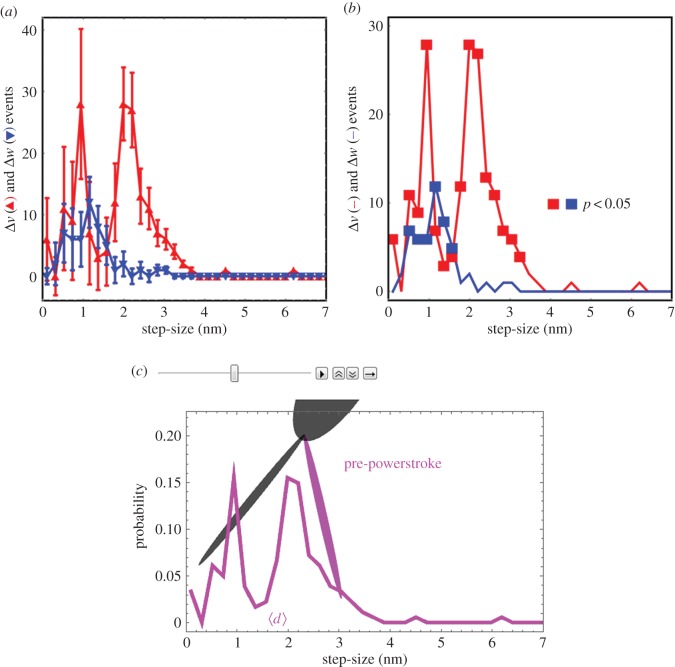
Panel *a* shows the step-size histogram residual with standard deviation errors for the pre-powerstroke residual (Δ*v*) and time-uncorrelated relaxed state (Δ*w*). Panel *b* is the same as panel *a* without error bars and with points on the curve that are significantly different from 0 indicated by the solid square symbol. Red indicates Δ*v* and blue indicates Δ*w*. Panel *c* is one frame from a video representing the active cycle of the myosin lever-arm using observations from active, relaxed and rigor states of the *in vivo* zebrafish muscle. The entire video is available in electronic supplementary material (movie S6, cycle.avi).

Transitions between time-sequenced coordinates correspond to a step-size using equation (2.4). Figure 13 shows the step-size histogram residual, Δ*v*, defined in equation (2.5) for the active state (red). We also computed the residual for a suitable control consisting of relaxed single myosin images uncorrelated in time. The time-uncorrelated relaxed state residual, Δ*w* (blue), uses equation (2.5) but with *w*_control_, the step-size distribution for the time-uncorrelated relaxed data, replacing *v*_ac_. Time-uncorrelated relaxed state data do not appear in *v*_re_. Panel *a* indicates the event number average and standard deviation for Δ*v* versus populated step-size domain, for the active state with solid red triangles and red error bars, and for the control data (*w*_control_) with solid blue inverted triangles and blue error bars. Panel *b* indicates the same data without error bars and where solid red or blue squares identify data points significantly different from 0 (*p*-value using one-way ANOVA). Above the 1.5 nm step-size interval, only the active state has significantly populated step-size probability with periodic residual enhancements corresponding to discrete *in vivo* active step-sizes at approximately 2, approximately 3 and more than 4 nm essentially as observed previously in permeabilized cardiac muscle fibres [11].

Above the 1.5 nm step-size interval, the part of Δ*v* significantly more than 0 corresponds to approximately 17% of the active steps. The Δ*w* in this region has no significant probability, indicating the time-uncorrelated data contain no step-size probability not already represented by the *v*_re_ and *v*_ri_ basis vectors. Below the 1.5 nm step-size interval, both *v*_ac_ and *w*_control_ have significant probability density unrepresented by the *v*_re_ and *v*_ri_ basis vectors. Most event density in the basis vectors and *w*_control_ resides in this domain, but the part of Δ*w* significantly more than 0 corresponds to just 6% of all events in the control (time-uncorrelated relaxed) data. This represents an upper limit on potentially misplaced lever-arm rotation assignments using the residual method in equation (2.5).

The Δ*v* indicates that the active muscle has largest overlap with relaxed muscle. Event breakdown has the *v*_ac_ basis vector containing 686 events, with 116 of them identified with pre-powerstroke lever-arm rotation, 506 with relaxed lever-arms and 64 with lever-arms in rigor. It corresponds to a steady-state distribution in the active isometric state of 17%, 74% and 9% of the cross-bridges in pre-, relaxed and post-powerstroke states. The *v*_re_ and *v*_ri_ basis vectors contain 412 and 241 events, respectively, whereas *w*_control_ has 123 events. Figure 13*c* represents a lever-arm in the pre-powerstroke conformation corresponding to the 3 nm step-size for the Δ*v* probability density (purple curve) identical to that in the other panels. Average step-size, 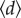, for Δ*v* is indicated above the horizontal step-size axis. Panel *c* is one frame from the video in the electronic supplementary material (movie S6) representing lever-arm movement over the whole cross-bridge cycle.

**Figure 14.**
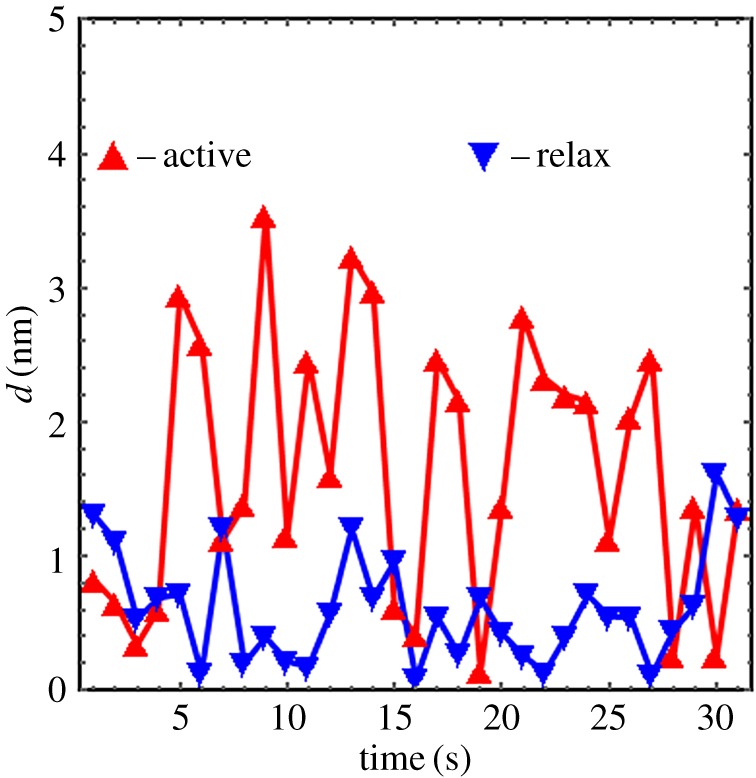
Real-time data comparing consecutive frame step-size *d* (equation (2.4)) for active and relaxed muscle. These data are a subset of the datasets providing the step-size histogram residual in figure 13. The active muscle has larger average *d* (1.6 versus 0.5 nm) and dispersion (1.0 versus 0.5 nm s.d.). The oscillation of *d* from active isometric muscle suggests a cycle time of approximately 4 s. If the sampling rate is too low (likely) then the observed oscillation is an alias for a higher-frequency oscillation.

Real-time data compare consecutive frame step-size *d* (equation (2.4)) for active and relaxed muscle in figure 14. The active muscle has larger average *d* (1.6 versus 0.8 nm) and dispersion (1.0 versus 0.4 nm s.d.). Real-time data sampling at 1 s per frame in the figure is (probably) too low to avoid aliasing time-dependent displacement but conclusively implies the active cross-bridge angular displacement usually exceeds that in relaxed muscle.

## 4. Discussion

Hypo- and hyper-contractility in cardiac and skeletal muscle associate with the effects of inheritable myopathies (IM), ageing and lifestyle choices. Inheritable cardiomyopathies frequently link to the myosin motor powering contraction. IM-linked mutations locate throughout the myosin, impacting each motor function. Despite the correlation between disease and the IMs impacting myosin, there is a persistent ambiguity between measurable molecular-level mutant myosin characteristics and IM phenotype. An approach addressing this challenging problem uses the visible light-transparent zebrafish embryo model for human muscle disease. It permits *in vivo* one-to-one registration of single myosin mechanical characteristics with disease phenotype. Here we characterize *in vivo* single myosin orientation and step-size and physiological performance of whole muscle in contracting skeletal muscle of a zebrafish embryo, merging bottom-up single molecule and top-down phenotypic myosin characterizations. *In vivo* single myosin step-size is also compared with its *in vitro* counterpart measured with the Qdot assay on zebrafish skeletal myosin. Contrasting *in vitro* and *in vivo* myosin characteristics elucidates the significance of the motor context.

Earlier *in vivo* work on zebrafish embryos studied the orientation of single HCRLC-PAGFP-tagged myosin cross-bridges in relaxed skeletal muscle [13]. It provided the key observations and methods for determining the GFP emission dipole orientation relative to the myosin lever-arm. The solved structure, depicted in figure 1, allows us to interpret observations from this myosin probe in terms of the lever-arm attitude. We now introduce the study of two additional live zebrafish physiological states in skeletal muscle. Steady-state isometric contraction is induced by strychnine treatment of the zebrafish and is a reversible perturbation of the cross-bridge orientation distribution under the conditions employed. The rigor (ATP-depleted) state in live trunk muscle was induced by a long-term strychnine treatment of the zebrafish. The relaxed (resting), rigor and active isometric state investigations cover the full *in vivo* physiological muscle cycle experienced by a single skeletal myosin lever-arm facilitating the goal to measure *in vivo* myosin step-size.

*In vivo* modification of gene expression developed the appropriate zebrafish phenotype for differing experimental requirements. Single molecule experimentation benefitted from sparse expression of the HCRLC-PAGFP in the mosaic pattern created by a transiently expressed gene. The sparse expression pattern reduced to a practical minimum the amount of contaminating background fluorescence that is critical to quantitative isolation of single molecule images. Physiological experimentation benefitted from robust expression of the HCRLC-GFP appropriately developed by transgenic zebrafish. Robust expression in this application corresponded to 23–38% total coverage of the skeletal myosin in the trunk muscle as implied by RT-PCR and SDS–PAGE. We find this reaches a practical threshold for reliable phenotypic characterization of the effect of myosin modification by HCRLC-GFP on isometric force in contraction. Embryo tension measurements on permeabilized WT and transgenic zebrafish combined with the estimates for skeletal myosin coverage by HCRLC-PAGFP implies the tag does not impair isometric contraction (figures 8–10 and tables 2 and 3).

*In vivo* characterization of lever-arm movement involves the recording of the fluorescence intensity images from single myosins in a sparsely photo-activated field of myosins from the zebrafish skeletal trunk muscle. Single myosins are identified by their quantized photoactivation and photobleaching events recorded in the time-correlated images (a movie). Single myosins have dipolar emission from their HCRLC-PAGPF tag that is interpreted at super-resolution for dipole orientation by recognizing the two-dimensional spatial distribution of the emitted light intensity [26]. Time-correlation is critical, because we interpret the time series of dipole orientations thus recoded as sometimes indicating the active lever-arm powerstroke. We distinguish between angular transitions involved in force generation (i.e. lever-arm rotation while myosin is strongly actin attached) versus random angular transitions owing to actin binding or unbinding events or movement while the cross-bridge is relaxed by comparing the active state angular transitions to those observed from relaxed and rigor states of the muscle. The residual of active angular transitions that cannot be emulated as a linear combination of angular transitions seen in the other states are powerstrokes. These powerstrokes, when interpreted as step-sizes by multiplying their rotation angle (in radians) by the lever-arm length, scale to 2–7 nm in length and occupy approximately 17% of the active angular transitions recorded. The dynamic active cycle of the myosin lever-arm, derived from the observations described here, is indicated in video S6 (cycle.avi) in the electronic supplementary material. These physiological states of the active cycle are correlated graphically with observed step-size and lever-arm orientation. It represents the first estimate for real-time and *in vivo* lever-arm dynamics in zebrafish skeletal muscle. One frame from the video depicting the pre-powerstroke orientation of the myosin lever-arm executing a 3 nm step is shown in figure 13*c*.

Canonical bottom-up investigation of single myosin cross-bridges *in vitro* has lately been accomplished in a motility assay using Qdot-labelled actin [7]. The Qdot assay measures the traditional single molecule mechanical attribute of motor step-size but in the context of an ensemble of actomyosin interactions. The ensemble context imposes a constant velocity constraint for the myosins interacting with one actin filament introducing the ‘second-characterization’ of the unitary step. In a myosin motor that produces multiple step-sizes, like the cardiac myosin [8], the second-characterization is step-frequency (*ω*) that adjusts longer step-size to lower frequency maintaining a linear actin velocity identical to that from a shorter step-size and higher-frequency actomyosin interaction. Here we have investigated the *in vitro* step-size and step-frequency of the zebrafish skeletal myosin for two reasons. First to understand the impact of the lever-arm bound HCRLC-GFP tag on myosin functionality, and second to explore complementarity of *in vitro* and *in vivo* step-size.

*In vitro* zebrafish skeletal myosin mechanical characterization involves Qdot assaying of step-size and step-frequency. We assayed native and exchanged myosin containing the pure ZRLC and the 70/30 mixture of ZRLC with HCRLC-GFP. Their step-size and step-frequency characteristics (and the *in vitro* motility velocity implied by these quantities) are similar (figure 7). These data likewise support the notion that the HCRLC-GFP tag does not impair contractility. Additionally, data of figure 7 show that the zebrafish skeletal myosin moves actin with three distinct step-sizes and step-frequencies that resemble those in cardiac myosin and different from the rabbit skeletal myosin [7]. Exchanged zebrafish myosin was obtained from adult fish, because this sample provided the needed quantity for myosin purification, hence the comparison with rabbit skeletal myosin could reflect species specificity rather than developmental differences.

We compare *in vitro* and *in vivo* myosin mechanical characteristics with step-size rather than step-frequency, because the *in vivo* active residual will be likely to add probability to the active step-sizes recognized in figure 13 with acquisition of higher time-resolution *in vivo* movies. The higher time-resolution should lead to more frequent isolation of powerstrokes, potentially altering the step-frequency (the area under the curves at discrete unitary step-sizes) but probably not the step-size. *In vivo* step-size differs from those observed *in vitro*, probably because their observation is under isometric contraction. They are similar to the discretized work production probabilities from single myosins observed in permeabilized cardiac papillary muscle fibres in isometric contraction [11]. The discrete work-producing steps when summed were attributed there to the larger powerstroke. This work indicates the same but now in the context of the *in vivo* muscle.

A novel *in vivo* method explores the transduction/mechanical-coupling algorithm-driving contractility from both bottom-up single molecule and top-down phenotypic perspectives, thus providing a penetrating assessment of the muscle motor conformation in contraction under *in vivo* conditions. We also define myosin's *in vitro* and *in vivo* complementarity in critically important metrics.

## Supplementary Material

Supplementary Material

## Data accessibility

Time-resolved images of single myosin emission patterns from active and relaxed muscle can be found in the electronic supplementary material.
