# Supplementary material for: In vivo myosin step-size from zebrafish skeletal muscle
Source: Open Biol. 2016 May 25;6(5):160075. doi: 10.1098/rsob.160075 (PMC4892436; doi:10.1098/rsob.160075)
Supplement: Supplementary Material [file rsob160075supp1.docx]

Supplementary Material

*In Vivo* Myosin Step-Size from Zebrafish Skeletal Muscle

Thomas P. Burghardt^1,2*^, Katalin Ajtai^1^, Naoko Takubo^1,3^, Xiaojing Sun^1^, and Yihua Wang^1^

^1^ Department of Biochemistry and Molecular Biology and ^2^ Department of Physiology and Biomedical Engineering, Mayo Clinic Rochester, Rochester, MN 55905

^3^ Present address: Department of Physiological Chemistry and Metabolism, Graduate School of Medicine, The University of Tokyo, 7-3-1 Hongo, Bunkyo-ku, Tokyo 113-0033, Japan


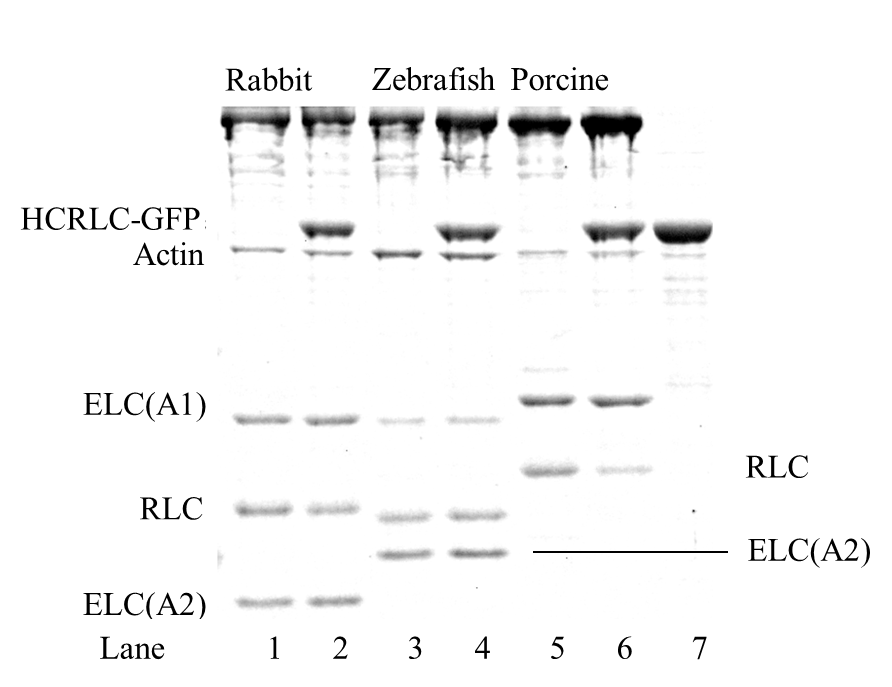


**Figure S1**. The SDS-PAGE of wild type and HCRLC-GFP exchanged myosins from: Rabbit skeletal (Lanes 1 & 2), zebrafish skeletal (Lanes 3 & 4), and porcine ventricular cardiac (Lanes 5 & 6). Lane 7 is HCRLC-GFP. The HCRLC-GFP replaced 34%, 30%, and 64% of the native RLC in rabbit skeletal, zebrafish skeletal, and porcine cardiac myosin, respectively.


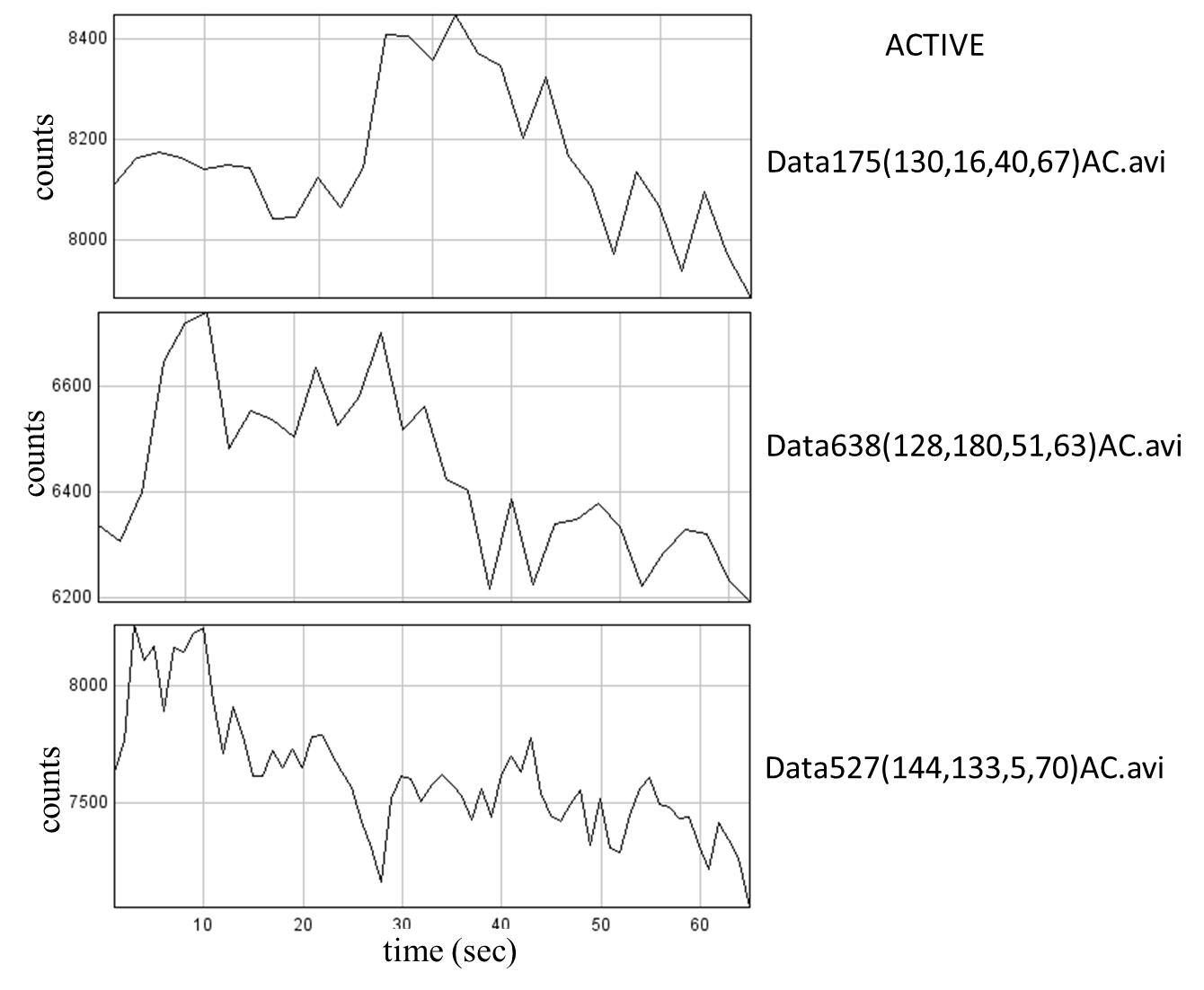


**Figure S2**. Raw GFP fluorescence vs time from RLC-PAGFP tagged myosin *in vivo* from active zebrafish embryo skeletal muscle under HILO illumination. It identifies single myosins by their quantized intensity change of 400-700 photons per second above background due to photoactivation and subsequent photobleaching to background. Counts indicate intensity integrated over the EMCCD camera 11x11 pixel array containing a photoactivated chromophore. Accompanying video files, with name listed next to the fluorescence vs time plots, contain the raw images.


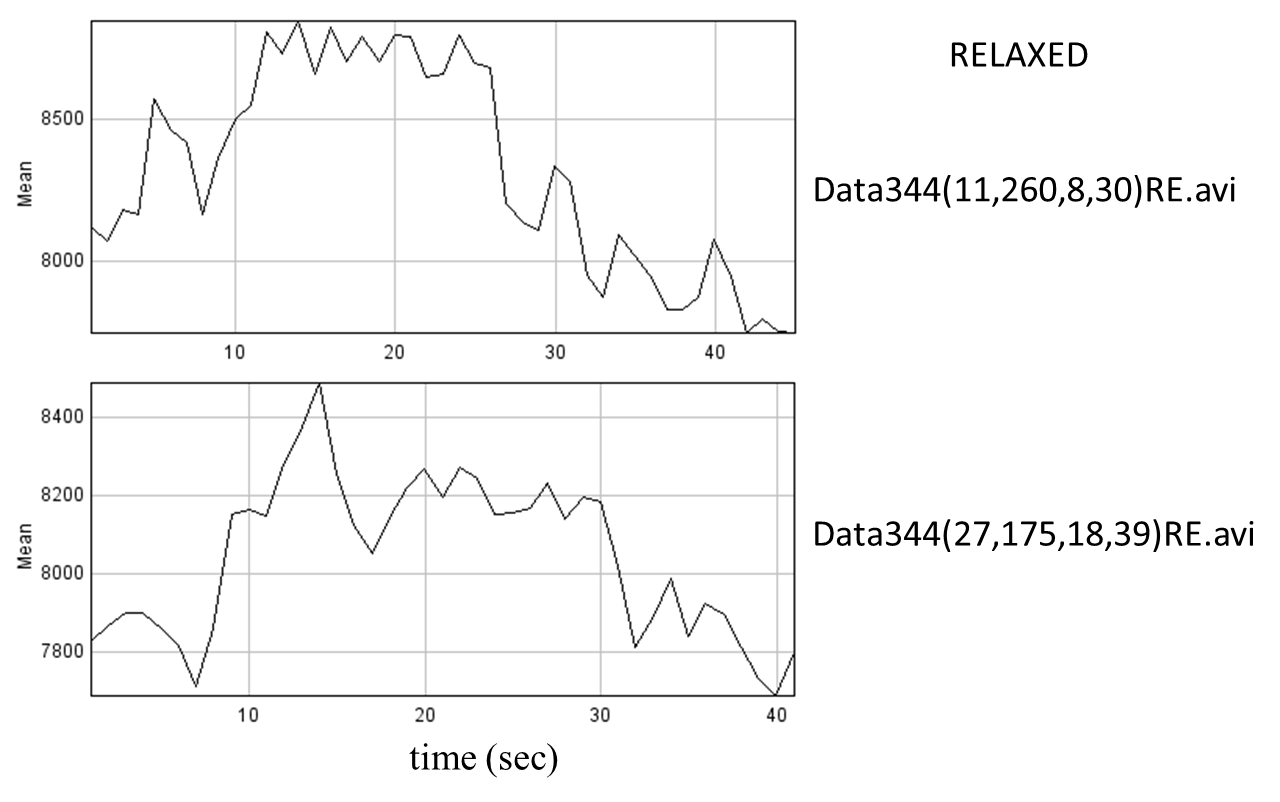


**Figure S3**. Same as **Figure S2** except for relaxed zebrafish embryo skeletal muscle. Accompanying video files, with name listed next to the fluorescence vs time plots, contain the raw images.
